# Comprehensive interrogation of CpG island methylation in the gene encoding COMT, a key estrogen and catecholamine regulator

**DOI:** 10.1186/1755-8794-7-5

**Published:** 2014-01-24

**Authors:** Theresa Swift-Scanlan, Christopher T Smith, Sabrina A Bardowell, Charlotte A Boettiger

**Affiliations:** 1 School of Nursing, University of North Carolina, Carrington Hall, CB #7460, Chapel Hill, NC, USA; 2 Lineberger Comprehensive Cancer Center, University of North Carolina, 450 West Drive, Chapel Hill, NC, USA; 3 Department of Psychology, University of North Carolina, Davie Hall, CB #3270, Chapel Hill, NC, USA; 4 Department of Psychology, Biomedical Research Imaging Center, and Bowles Center for Alcohol Studies, University of North Carolina, Davie Hall, CB #3270, Chapel Hill, NC, USA

## Abstract

**Background:**

The catechol-*O*-methyltransferase (COMT) enzyme has been widely studied due to its multiple roles in neurological functioning, estrogen biology, and methylation metabolic pathways. Numerous studies have investigated variation in the large COMT gene, with the majority focusing on single nucleotide polymorphisms (SNPs). This body of work has linked COMT genetic variation with a vast array of conditions, including several neurobehavioral disorders, pain sensitivity, and multiple human cancers. Based on COMT’s numerous biological roles and recent studies suggesting that methylation of the COMT gene impacts COMT gene expression, we comprehensively interrogated methylation in over 200 CpG dinucleotide sequences spanning the length of the COMT gene.

**Methods:**

Using saliva-derived DNA from a non-clinical sample of human subjects, we tested for associations between COMT CpG methylation and factors reported to interact with COMT genetic effects, including demographic factors and alcohol use. Finally, we tested associations between COMT CpG methylation state and COMT gene expression in breast cancer cell lines. We interrogated >200 CpGs in 13 amplicons spanning the 5’ UTR to the last exon of the CpG dinucleotide-rich COMT gene in *n* = 48 subjects, *n* = 11 cell lines and 1 endogenous 18S rRNA control.

**Results:**

With the exception of the CpG island in the 5’UTR and 1^st^ exon, all other CpG islands were strongly methylated with typical dynamic ranges between 50-90%. In the saliva samples, methylation of multiple COMT loci was associated with socioeconomic status or ethnicity. We found associations between methylation at numerous loci and genotype at the functional *Val*^*158*^*Met* SNP (rs4680), and most of the correlations between methylation and demographic and alcohol use factors were *Val*^*158*^*Met* allele-specific. Methylation at several of these loci also associated with COMT gene expression in breast cancer cell lines.

**Conclusions:**

We report the first comprehensive interrogation of COMT methylation. We corroborate previous findings of variation in COMT methylation with gene expression and the *Val*^*158*^*Met* genotype, and also report novel associations with socioeconomic status (SES) and ethnicity at several methylated loci. These results point to novel mechanisms for COMT regulation, which may have broad therapeutic implications.

## Background

Catechol-*O*-methyltransferase (COMT) enzyme has broad biological functions, principally the catabolism of biologically active or toxic catechols, including catecholamines and catecholestrogens [1]. As a result of its ubiquity, COMT has been implicated in a wide range of human conditions, including cancer [2], pain sensitivity [3,4], schizophrenia [5], affective [6], addictive [7,8], impulse control disorders [9], and Parkinson’s disease [10]. The clinical significance of these conditions has stimulated growing interest in COMT in recent decades, particularly following the discovery that COMT enzyme activity level in human tissues is genetically polymorphic, conferring low, intermediate, and high activities [11,12]. A great deal of research has focused on a common, functional, single nucleotide polymorphism (SNP) of COMT, *Val*^*158*^*Met* (rs4680), that is the most studied variant, due to its location within the exon 4 coding region. Specifically, the substitution of a methionine (Met) for a valine (Val) at position 158 results in three- to four-fold reduced activity of the COMT enzyme due to reduced protein stability [13-15]. COMT is the primary regulator of dopamine clearance in extrastriatal brain regions, including the prefrontal cortex [16-18], which has helped to motivate research into associations of *Val*^*158*^*Met* with neuropsychiatric disorders since the mid-1990’s [19]. Early neuroimaging studies found the COMT 158^Val^ allele to be associated with impaired prefrontal cognition and physiology, which could contribute to schizophrenia risk [5], and to modulate pain perception and brain responses to pain [4]. However, observed associations between various conditions and the 158^Val^ allele are modest and often inconsistent. Thus, more recent work has identified COMT haplotypes that are associated with more profound changes in COMT activity, in part by effects on COMT protein expression [20,21].

Finally, other studies have tried to shed light on additive contributions to disease state by considering COMT polymorphisms in combination with other polymorphic gene loci. For example, genotype × genotype analyses involving COMT *Val*^*158*^*Met* with the methylenetetrahydrofolate reductase (MTHFR) C^677^T polymorphism have shown strong interactive effects associated with elevated total plasma homocysteine in a case control study of elders with and without dementia [22] as well as on dopamine signaling [23], executive function [24], and cognition [25] in persons with schizophrenia. COMT × MTHFR and other multigene interactions have also been explored in breast cancer [26-28].

In addition to the reported genetic effects on COMT expression, activity, brain function, and associations with behavior or disease risk, several non-genetic factors have been reported to impact COMT function, either in isolation or via interactive effects on genetic associations. These include an age-dependent rise in COMT activity in the prefrontal cortex [29], interacting effects of *Val*^*158*^*Met* genotype and age on impulsive choice [30], and sexually dimorphic associations with COMT in humans [31,32].

In addition, numerous studies have reported interactions between *Val*^*158*^*Met* genotype and environmental stressors, impacting everything from prefrontal function [33], and affect-modulated startle [34], to risk of alcoholism [35], posttraumatic stress disorder [36,37], and impulsive aggression [38]. How these non-genetic factors modulate COMT effects is largely unknown, but epigenetic regulation at the level of DNA methylation is one potential mechanism. In fact, investigations of the association between alcohol use and DNA methylation is a rapidly expanding area of research, although no studies to date have specifically investigated methylation within the COMT gene, beyond a few CpGs [39].

COMT polymorphisms have been broadly explored, not only within neurobiology, but also for their role in carcinogenesis, particularly in hormonally distinct cancers of the uterus and breast [2,31,40]. As in multiple neurobiobehavioral studies, the associations between COMT *Val*^*158*^*Met* and breast cancer risk have been inconsistent or modest [41-43]. One meta-analysis of COMT *Val*^*158*^*Met* based on more than 30,000 cases and 38,000 controls, found an increased risk for breast cancer only when the sample was stratified by race [44]. Given the predominance of estrogen receptor negative breast cancer in African American women, it is plausible that the He *et. al.* (2012) finding may more precisely reflect varied COMT influences that are dependent on the estrogen receptor status of the tumor.

The rationale for studying the influence of COMT on hormonally influenced cancer relates to the role of COMT on catecholamines and in estrogen metabolism. Specifically, the catecholamine neurotransmitters dopamine, norepinephrine, and epinephrine are synthesized primarily in the adrenal glands, and are derived metabolically from the amino acids tyrosine and phenylalanine [45]. The COMT enzyme effects the degradation of both catecholamines and catecholestrogens (important intermediary metabolites in estrogen induced cancers), by the addition of a methyl group [46]. The methyl (CH3) group with which COMT carries out targeted destruction of catechol compounds is provided by S-adenosyl methionine (SAM), a key methyl donor in the folate metabolic pathway with a pivotal role in epigenetic alterations in general, [45] and in epigenetic changes in cancer in particular [47,48].

Past studies have focused on genetic variations, particularly SNPs as vulnerability factors for cancer [40,44], schizophrenia [5], pain [49], emotional processing [50], and broad cognitive functioning [51]. While the discovery of COMT genetic polymorphisms has illuminated a host of biologic vulnerabilities ranging from cancer to neurobehavioral pathology, recent evidence suggests that epigenetic changes, particularly DNA methylation of CpG (e.g., “C – phosphate - G” on the same DNA strand) dinucleotide sequences in the COMT gene, may also have an important impact on COMT function [52,53]. DNA methylation may be inherited (via imprinting for example), and/or may result from somatic changes due to a wide array of environmental influences, such as exposure to stress [54], diet, alcohol and tobacco use [47,48]. Once effected, somatic DNA methylation changes are perpetuated in successive cell generations during cell replication and division [55]. DNA methylation can affect gene transcription via interactions with DNA packaging chromatin proteins, and/or by interfering with the binding of enhancers, transcription factors, or other proteins involved in transcriptional regulation [15]. In such cases, DNA methylation may exert its effects either synergistically or independent of known genetic variations.

Based on recent findings showing evidence of differential COMT DNA methylation [56], and associations between COMT methylation state and vulnerabilities to a spectrum of conditions ranging from schizophrenia [57,58] to the cognitive effects of stress [33], we sought to provide the first comprehensive assessment of DNA methylation throughout the COMT gene, simultaneously considering *Val*^*158*^*Met* genotype in association with methylation changes. We hypothesized that differential methylation (DM) would be associated with demographic factors, reflecting a possible environment × gene interaction, and with variation in COMT genotype, which would support COMT allele-specific methylation. Moreover, we hypothesized that different levels of alcohol use would also be associated with COMT methylation, possibly in a COMT genotype-dependent manner. We tested these hypotheses using saliva samples collected from a non-clinical community sample of adult social drinkers (ages 18–40) with no known history of neurologic or psychiatric illness, and a spectrum self-reported alcohol use. The epigenetic variation in peripheral COMT detected in DNA derived from human saliva has been shown to be similar to those found in COMT in the brain [58]. In addition, we were able to test for associations between COMT DM and observed variation in COMT gene expression in breast cancer cell lines. We interrogated over 200 CpGs in 13 amplicons spanning the 5’ UTR to the last exon of the CpG dinucleotide-rich COMT gene in *n* = 48 subjects, *n* = 11 cell lines and 1 endogenous 18S rRNA control. We report DM at multiple COMT loci in association with *Val*^*158*^*Met* genotype, socioeconomic status (SES), ethnicity, alcohol use, and gene expression.

## Methods

### Sample characteristics

Participants (*n* = 48) were recruited from the University of North Carolina, Chapel Hill (UNC) and surrounding community. Participants were healthy individuals 18–40 years old with no known past or present neurological or psychiatric diagnoses, no history of substance use disorders, and no current use of psychoactive medications or other psychoactive substances aside from moderate caffeine, nicotine or alcohol. All subjects were native English speakers, had at least a high-school education, and reported having consumed alcohol at least once in their lifetime. Information regarding participants’ occupation and education was collected via a questionnaire and quantified based on the method of Hollingshead [59]. Participants gave written informed consent, as approved by the UNC Office of Human Research Ethics.

### General procedure

Participants completed questionnaires to allow quantification of SES according to Hollingshead [59]. We also collected data regarding each participant’s alcohol use via the Alcohol Use Disorders Identification Test [60].

### Saliva samples: DNA extraction and genotyping

COMT*Val*^*158*^*Met* (rs4680) genotyping was performed on DNA extracted from saliva samples (DNA Genotek, Kanata, Ontario, Canada) using TaqMan technology (Life Technologies, Foster City, CA), as described previously [61]. Genotyping was performed by the Duke Center for Human Genetics. Allele frequencies in this sample did not deviate from Hardy–Weinberg equilibrium (*x*^*2*^ = 1.91, *df* = 1, *p* = 0.17).

### Sodium bisulfite conversion of DNA

Like many methylation assays, the EpiTYPER assay hinges on an initial PCR reaction using primers specific to sodium bisulfite (NaBi) converted DNA, wherein all un-methylated cytosines are converted to uracil, then subsequently to thymine during PCR. Conversely, methylated cytosines (e.g., CpG dinucleotides) are not converted, and thus allow assessment of the true methylated state of the interrogated loci prior to NaBi conversion. The EZ DNA Methylation-Direct Kit (Zymo Research, Irvine, CA, USA) was used to sodium bisulfite convert genomic DNA extracted from cell lines or saliva samples. Sodium bisulfite conversion was performed on a thermocycler at 95°C for 30 s and 50°C for 15 min for twenty cycles as per protocol.

### Quantifying COMT methylation using the EpiTYPER MassARRAY Platform

**Figure 1:**
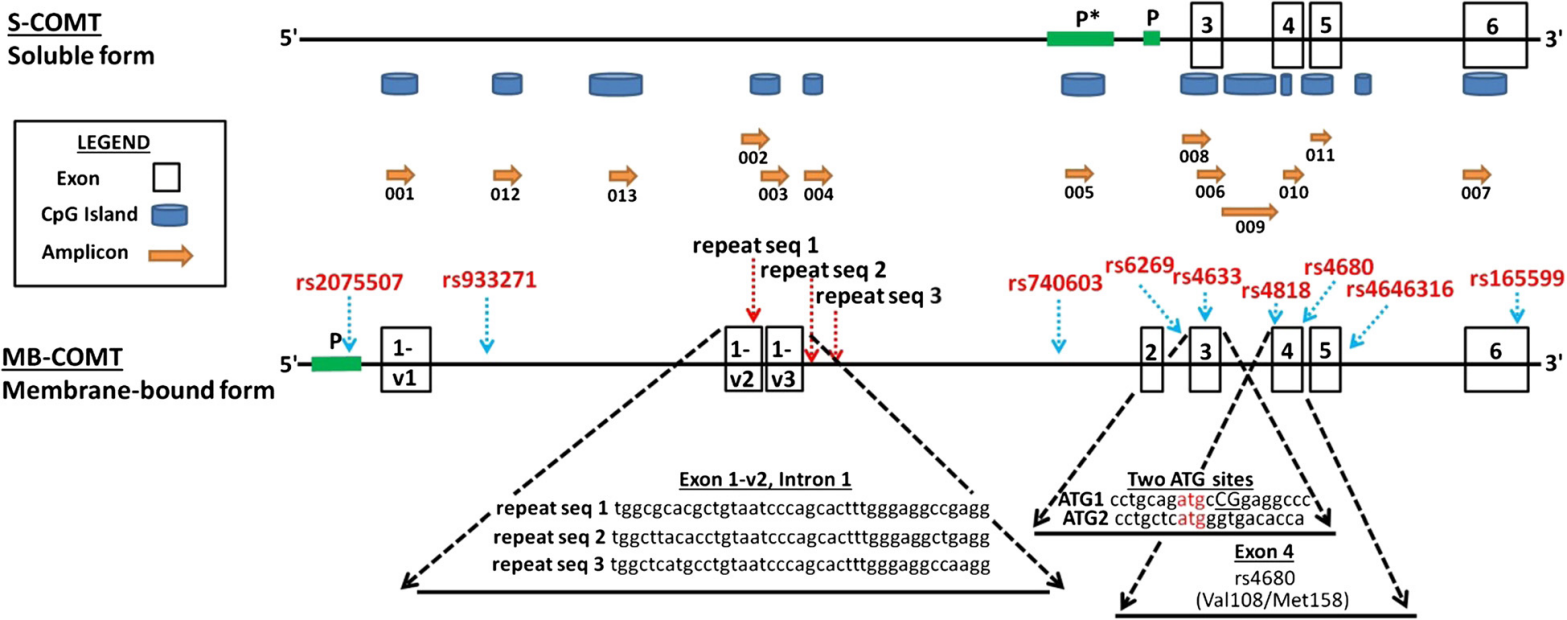
**COMT gene features relative to amplicons interrogated for methylation.** COMT spans ~32 kb (chromosome 22q11.21, human build NCBI 37/hg 19, bp 19925733 – 19957832), with two major transcript variants encoding soluble and membrane bound (S-COMT and MB-COMT) enzyme forms, respectively (drawn to scale). 13 amplicons (001–013, orange arrows), were designed to interrogate methylation in CpG islands (blue cylinders) throughout COMT. Previously identified SNPs associated with impulsivity or pain sensitivity are listed by rs number (red font), with approximate SNP position indicated by blue vertical arrows. Top row: The S-COMT variant is encoded by four exons (black lined white boxes), regulated by promoter P1 (green bar), and possibly by a predicted promoter (PP) 5’ of P1. Bottom Row: The MB-COMT is encoded by 6 exons (with multiple possible variants of exon 1 (1-v1, 1-v2, 1-v3). Expanded view (dashed diagonal lines), highlight three ~40 bp repeat sequence motifs that we identified spanning ~1.5 kb beginning in exon 1-v2 through intron 1; the ATG1 and ATG2 translational start sites (TSS) in Exon 3 for S-COMT and MB-COMT, respectively; and the rs4680 SNP in Exon 4 encoding the substitution of valine (val) with methionine (met) at codon 158.

Percent methylation was quantified throughout the COMT gene using mass spectrometry with the EpiTYPER^®^ T complete reagent kit as previously described [62]. The SEQUENOM EpiTYPER^®^ methylation assay has been validated in numerous studies and previously described in detail [63-65]. We custom designed primer sets specific for sodium bisulfite converted DNA for 13 amplicons spanning 5’ of exon 1 to the 3’ end of COMT located in exon 6 (Figure 1, Table 1). The 13 amplicons were chosen because they either assayed a CpG island as defined by a CpG content greater than 50% spanning 300 bp or more, and/or because they allowed interrogation of CpG sequences located in important regulatory COMT gene regions.

**Table 1:** COMT amplicon description

**Amplicon name**	**Human build 37**	**Length**	**CpG**
	**Position, Chr. 22**	**BP**	**Coverage**
COMT_001	19928951	400	37
COMT_002	19938388	418	17
COMT_003	19938762	371	13
COMT_004*	19940043	226	8
COMT_005	19946527	438	20
COMT_006	19949994	392	16
COMT_007*	19955930	379	14
COMT_008	19949536	484	11
COMT_009*	19950497	405	11
COMT_010	19951020	277	14
COMT_011*	19951513	455	11
COMT_012*	19931875	379	17
COMT_013	19935171	394	14

COMT amplicons 001 – 013 are listed together with their human build NCBI 37/hg 19 bp location on chromosome 22. Amplicon size (in bp), together with the number of CpGs interrogated for methylation are provided. *amplicons were not differentially methylated, or could not be reliability amplified due to repetitive DNA sequence in the region (COMT_004, see Figure 1).

PCR of all 13 COMT amplicons was carried out on 10 ng of NaBi converted genomic DNA in 5 μl reaction volumes under the following conditions: 95°C for 2 min, with a series of touch down reactions for 2 cycles at 95°C for 30 s, 60°C for 30 s, 72°C for 1 min, 2 cycles each with a 59°C, 58°C and 57°C annealing, respectively, followed by 40 cycles at 56°C annealing. The PCR product (2 μl) was added to 5 μl of the T-Cleavage reaction as a template for the *in vitro* transcription reaction as per the EpiTYPER protocol. The final T cleavage reaction produced un-methylated and methylated CG containing fragments that were resolved and quantified for percent methylation via mass spectrometry. We discovered that COMT_004 would not amplify efficiently due to stuttering of the Taq polymerase through flanking repetitive sequences in the region (Figure 1), and it was therefore excluded from further analyses. Similarly, COMT_007, 009, 011 and 012 were not differentially methylated in our initial sample set and therefore were not included in subsequent analyses.

### Cell line gene expression

Breast cancer cell lines were cultured as described previously [62]. Gene expression in breast cell lines was quantified using qRT-PCR on a 7500 Real-Time PCR Platform (Life Technologies, Foster City, CA). Relative cDNA quantity was measured using pre-designed ABI TaqMan probes and primers for COMT (COMT-Hs00241349_m1) and 18S rRNA (18S rRNA -4333760-1007035_g1) as the endogenous expression control (Life Technologies). Cell line cDNAs were examined in triplicate, with qRT-PCR cycling as follows: 50°C for 2 min, denaturation at 95°C for 10 min, followed by 40 cycles of 95°C for 15 s, and 60°C annealing for 1 min. The only commercially available COMT TaqMan probe does not distinguish S and MB transcript variants.

*Statistical Analyses* Values reported as mean ± SEM, unless otherwise stated. Statistical analyses performed in SPSS (IBM Corp., Armonk, NY), except where otherwise noted.

### Hierarchical clustering analysis

**Figure 2:**
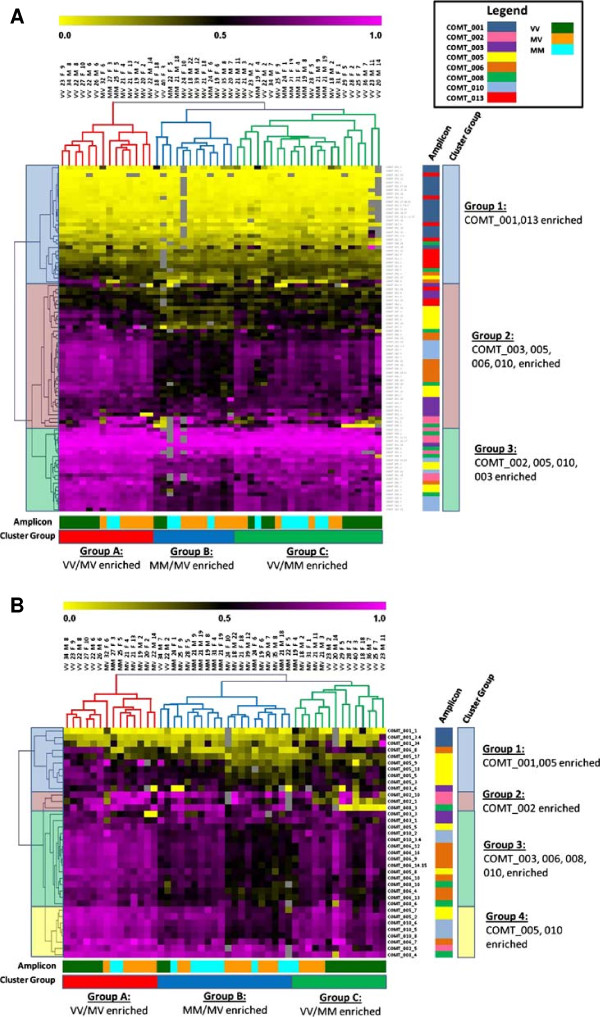
**Hierarchical clustering analysis of COMT methylation in the UNC dataset.** The clustergram is highlighted on the left to display the major clada or related groups of methylated CpGs for each dataset. Percent methylation is represented on a color continuum of bright yellow (0%), black (50%) to bright purple (100%). Colored bars on the right of the clustergram display the amplicon members of each group. (COMT_001 = *dark blue*, COMT_002 = *salmon*, COMT_003 = *purple*, COMT_005 = yellow, COMT_006 = *purple,* COMT_008 = *green,* COMT_010 = *light blue,* COMT_013 = *red*). Unsupervised hierarchical clustering analysis (HCA) by CpG unit of **A** 48 subjects and 110 CpGs shows 3 groups of subjects by COMT *Val*^*158*^*Met* genotype, age, sex, and alcohol use (AUDIT score). Group A is enriched for VV + MV genotypes, Group B for MM + MV genotypes and high AUDIT scores, and Group C for VV + MM genotypes. **B** Clustergram reveals the 39 most differentially methylated CpGs after applying a 40% standard deviation filter, after which Group C becomes predominantly VV genotype enriched.

We first performed unsupervised hierarchical clustering using MeV (version 4.8.1) of the TM4 software suite [16] with complete linkage, Euclidean distance parameters selected for COMT percent methylation values in the UNC dataset (Figure 2A). In order to identify the most differentially methylated CpGs in COMT amplicons 001, 002, 003, 005, 006, 008, 010, and 013, we applied a 40% standard deviation filter with the MeV application (Figure 2B).

### Robust partial correlation analysis

To eliminate concerns regarding violation of parametric test assumptions, bootstrapping procedures (*n* = 1000 resamples) were used in tests of statistical significance. We calculated partial correlation Pearson coefficients in models for demographic factors and COMT methylation. To evaluate the relationship between COMT methylation and gene expression in cell lines, we calculated Spearman’s *rho* correlations due to the skewed distribution of gene expression data. Correction of *p*-values for multiple comparisons using the false discovery rate (FDR) procedure [66] was carrying out in R (http://www.R-project.org).

## Results

### Sample characteristics

Healthy participants ages 18–40 (*n* = 48) provided a saliva sample from which DNA was extracted for *COMT Val*^*158*^*Met* genotyping and COMT gene methylation analysis. The sample was well educated and predominately white, and was balanced in terms of sex and light or heavy social drinking (see Table 2).

**Table 2:** Demographic data

	**(*n*** **= 48)**
Age (yrs)	25 ± 6
Education (yrs)	16 ± 2
SES	45 ± 10
Sex (% female)	52.1
Ethnicity (% white)	72.9
Black (%)	12.5
Hispanic (%)	4.2
Asian (%)	6.3
Other/mixed (%)	4.2
AUDIT score	7.9 ± 5.4

Values are reported as mean ± standard deviation. AUDIT, Alcohol Use Disorders Identification Test; SES, socioeconomic status.

### Characterization of methylation sites within the COMT gene

Figure 1 highlights the COMT gene structures which encode the two major protein variants; the soluble (S-COMT) and membrane-bound (MB-COMT) forms, respectively. Table 1 describes the specific location and relevant features for the 13 amplicons interrogated. We performed unsupervised hierarchical clustering analysis (HCA) on percent methylation values for CpGs throughout COMT based on Euclidean distance and complete linkage mapping (Figure 2A). In order to identify the most differentially methylated CpGs, we applied a standard deviation (SD) filter of 40% and obtained the most informative CpGs for the UNC dataset (Figure 2B). HCA of DNA methylation throughout COMT revealed 3 distinct groups or clada relative to the Val/Met genotypes (Val/Val + Met/Val mix, Met/Met + Met/Val mix, and a Val/Val enriched group). With the exception of the CpG island in the 5’UTR and 1^st^ exon, all other CpG islands were strongly methylated with a dynamic range of methylation typically between 50-90%.

**Figure 3:**
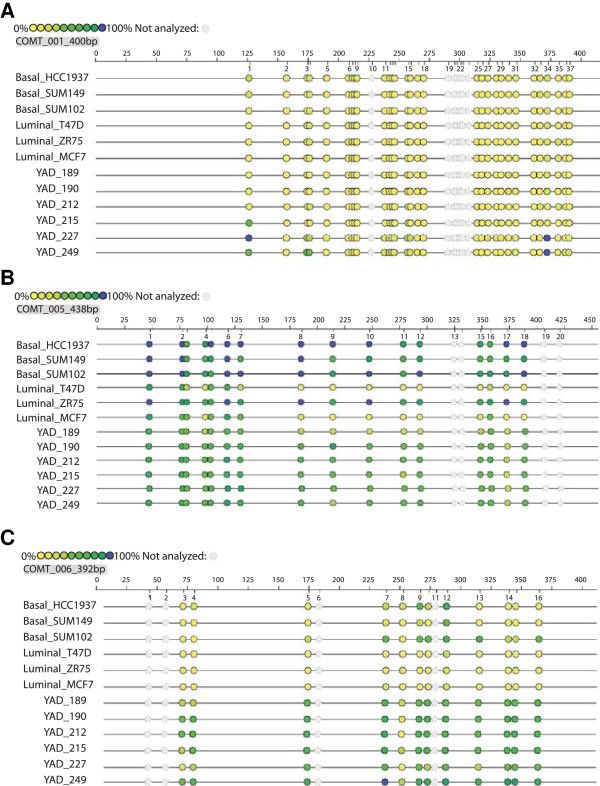
**Epigrams of amplicons illustrate variable methylation by sample type.** The Sequenom EpiTYPER MassARRAY platform was used to calculate percent methylation for CpG dinucleotides within each of the three amplicons shown. The epigram schematic illustrates percent methylation for each consecutive CpG (colored circle) on a continuum from yellow (0%) to navy blue (100%). Shaded circles represent CpGs that could not be quantified because they fell outside the Mass Dalton allowable detection window of the EpiTYPER software. Twelve samples per amplicon are shown to illustrate differential methylation: Rows 1–6 represent two groups of biologically distinct breast cancer cell lines (three basal-like and three luminal breast cancer cell lines respectively). Rows 7–12 are from six different healthy saliva samples. **A.** Amplicon COMT_001 is located in the CpG island 5’ and including exon 1 (Figure 1) and is relatively hypomethylated, with CpGs 1,3,4, and 34 showing the most heterogeneity across sample type. **B.** Amplicon COMT_005, located in the CpG island within the predicted promoter (P*) for S-COMT, is relatively hypermethylated for CpGs 1–7 for all samples, and most differentially methylated between CpGs 8–18 as illustrated by breast cell line samples T47D & MCF7, & saliva sample YAD189. **C.** Amplicon COMT_006, is located in Exon 3 (Figure 1), and like COMT_005, is homogeneously methylated at the beginning of the amplicon (CpGs 1–6), with differential methylation occurring between CpGs 7–16, as evidenced by relative hypermethylation of these loci in the basal versus the luminal cell lines.

Figures 3A- 3C are “epigrams” which serve to illustrate percent methylation variance per CpG unit across three selected amplicons; COMT_001, 005 and 006. These amplicons were chosen because they showed CpG unit methylation variability across sample types. As seen in Figure 3A, COMT_001 is relatively hypomethylated (with colored circles in the yellow continuum indicating percent methylation values of 20 percent or less), for breast cancer cell lines, and for three of the saliva samples. Differentially methylated CpG units, when they occur, are observed for CpGs 1, 3, 4, and 34 in the COMT_001 amplicon (Figure 3A). Conversely, COMT_005 and 006 (Figures 3B- 3C), are relatively hypermethylated. Notably, both COMT_005 and 006 are differentially methylated between biologically distinct basal and luminal breast cancer cell lines. Moreover, methylation variance, when and if it occurs, begins well into the amplicon at CpG 8 or 7 for the 005 and 006 amplicons, respectively. COMT DNA Methylation derived from saliva samples from healthy adults display a wholly different pattern from cell lines, with CpGs 8–17 for amplicon 005, and CpGs 3, 4, 8, 10 and 13 for amplicon 006 showing the most percent methylation variability. Note that in 2005, Murphy and colleagues interrogated 6 CpGs that fall within our amplicons 006 and 008, finding that our COMT_006, CpG #3 (Figure 3C) was the most differentially methylated in a mixed sample of patients with schizophrenia and unaffected siblings [56].

### Factors associated with differential methylation of the COMT gene

Percent methylation of CpG units within the COMT gene by individual sample characteristics is presented in Table 3. Within the full sample, no significant differences in COMT methylation were found to associate independently with age, sex, or alcohol use, when controlling for effects of other factors, which also included COMT *Val*^*158*^*Met* genotype, SES, and ethnicity. Relative to non-white participants, whites had significantly greater methylation at CpG 003_1 (*r* = .421, 95% CI: .103, .696, *p* = .008). Whites also had significantly less methylation at CpG 006_3 (*r* = -.336, 95% CI: -.644, .051, *p* = .039), which is located in exon 3, the exon containing both ATG translational start sites for S- and MB-COMT variants, and the locus previously reported to show high DM across individuals [56]. We also observed a significant negative correlation between SES and methylation at CpG 001_34 (*r* = -.331, 95% CI: -.653, .044, *p* = .049). In contrast to these sporadic and mostly weak effects, we found that numerous CpG’s within the COMT gene were DM by genotype at the *Val*^*158*^*Met* (rs4680) SNP, with all but one (008_3) showing reduced methylation with increasing numbers of 158^Met^ alleles. CpGs within amplicons 005 and 006 were particularly affected, with additional sites in amplicons 008 and 010. Notably, two of our observed correlations survived FDR correction for multiple comparisons (alpha = .05): 006_8, and 008_3. As seen in Figure 1, these amplicons fall within a region of the gene dense with CpG islands, with amplicon 010 including the *Val*^*158*^*Met* SNP (Figure 1).

**Table 3:** COMT methylation in healthy human subjects versus individual factors

**CpG Units**	**Age**	**Sex**^**a**^	**AUDIT score**	**Ethnicity**^**b**^	**SES**	**COMT**^**Met **^**alleles**
***r (95% CI) p***						
001_1	-.099 (-.401, .390) .565	.071 (-.397, .357) .681	-.021 (-.461, .381) .905	-.124 (-.556, .247) .470	.039 (-.236, .262) .821	.003 (-.381, .474) .988
001_3.4	-.022 (-.241, .292) .901	-.051 (-.331, .313) .766	-.085 (-.411, .336) .612	-.187 (-.454, .216) .274	-.137 (-.546, .164) .425	-.084 (-.322, .331) .624
**001_34**	.037 (-.221, .355) .830	-.081 (-.352, .243) .639	-.206 (-.543, .110) .227	-.201 (-.568, .166) .239	-.331 (-.653, .044) .049*	-.018 (-.290, .336) .919
001_avg	-.029 (-.256, .265) .865	-.034 (-.348, .223) .845	-.169 (-.482, .158) .323	-.235 (-.520, .146) .168	-.232 (-.521, .065) .174	-.037 (-.358, .343) .830
002_1	-.051 (-.373, .284) .768	.058 (-.258, .415) .737	.038 (-.263, .386) .825	.195 (-.134, .528) .256	-.189 (-.479, .124) .269	.130 (-.214, .454) .451
002_5	.073 (-.382, .440) .673	.168 (-.161, .529) .326	.038 (-.270, .338) .826	-.066 (-.386, .311) .701	-.235 (-.542, .117) .168	.282 (-.090, .600) .096
002_10	.002 (-.348, .382) .991	.081 (-.222, .434) .638	-.044 (-.351, .278) .797	.145 (-.208, .498) 398	-.222 (-454, .046) .194	.257 (-.049, .514) .130
002_avg	-.009 (-.336, .338) .960	.097 (-.195, .463) .574	.009 (-.307, .324) .959	.140 (-.208, .494) 415	-.230 (-.470, .092) .177	.227 (-.100, .495) .184
**003_1**	-.118 (-.461, .229) .479	-.199 (-.484, .137) .231	-.052 (-.334, .230) .758	.421 (.103, .696) .008**	-.081 (-260, .412) .665	.138(-.182, .423) .408
003_3	-.154 (-.415, .131) .363	.165 (-.152, .467) .329	-.099 (-.379, .222) .561	-.278 (-.520, .008) .096	.074 (-.260, .412) .665	-.013 (-.376, .308) .940
003_6	.135 (-.128, .387) .433	-.188 (-.453, .120) .272	.151 (-.146, .443) .379	-.162 (-.396, .112) .344	-.223 (-.435, .046) .190	-.092 (-.450, .291) .593
003_avg	.031 (-.301, .306) .853	-.107 (-.420, .182) .522	.061 (-.252, .339) .717	-.049 (-.390, .311) .769	-.187 (-.509, .209) .260	-.013 (-.359, .352) .937
**005_2**	-.239 (-.555, .214) .148	.028 (-.327, .356) .866	-.159 (-.434, .156) .341	-.209 (-.503, .184) .208	.041 (-.261, .366) .806	-.333 (-.596, -.063) .041*
005_3	-.065 (-.343, .273) .700	-.125 (-.422, .201) .454	-.116 ( -.391, .203) .489	-.159 (-.447, .176) .340	-.091 (-.358, .209) .587	-.193 (-.513, .128) .247
005_5	-.127 (-.416, .236) .446	.039 (-.283, .378) .815	-.068 (-.401, .262) .685	-.144 (-.438, .197) .388	-.136 (-.398, .202) .414	-.317 (-.592, -.047) .053
005_7	-.147 (-.451, .229) .380	-.120 (-.403, .216) .922	-.072 (-.354, .229) 668	-.176 (-.444, .184) .291	-.099 (-.400, .216) .553	.265 (-.547, .039) .107
**005_8**	-.103 (-.407, .280) .540	-.016 (-.349, .327) .922	-.120 (-.405, .192) .474	-.234 (-.523, .142) .157	-.074 (-.351, .264) .657	-.359 (-.601, -.072) .027*
005_9	-.058 (-.405, .381) .731	-.029 (-.342, .324) .863	-.207 (-.448, .117) .213	-.196 (-.511, .146) .239	.018 (-.329, .332) .914	-.077 (-.374, .160) .646
**005_10**	-.059 (-.381, .298) .723	-.064 (-.388, .276) .701	-.103 (-.416, .238) .539	-.276 (-.535, .084) .094	.120 (-.177, .431) .472	-.403 (-.632, -.108) .012*
**005_17**	.038 (-.230, .336) .819	-.193 (-.496, .172) .245	-.144 (-.418, .135) .389	-.290 (-.551, .030) .078	.086 (-.241, .413) .607	-.383 (-.613, -.137) .018*
**005_18**	-.022 (-.310, .327) .897	-.181 (-.471, .153) .284	-.203 (-.478, .131) .229	*-*.345 (-.623, -.026) .037*	.007 (-.297, .305) .968	-.379 (-.585, -.123) .021*
**005_avg**	-.098 (-.414, .299) .558	-.083 (-.398, .234) .621	-.161 (-.435, .152) .333	-.257 (-.542, .099) .119	-.017 (-.358, .336) .921	-.325 (-.588, -.049) .046*
006_3^c^	-.031 (-.357, .241) .856	-.037 (-.364, .320) .823	-.056 (-.347, .256) .738	-.336 (-.644, .051) .039*	.158 (-.200, .480) .342	.013 (-.333, .309) .937
006_4	-.027 (-.333, .259) .873	-.099 (-.412, .263) .554	-.094 (-.242, .318) .774	-.242 (-.529, .131) .143	.152 (-.166, .567) .363	-.093 (-.410, .225) .580
006_7	-.060 (-.365, .221) .722	.079 (-.241, .392) .638	.048 (-.242, .318) .774	-.179 (-.433, .165) .282	.122 (-.144, .361) .467	-.198 (-.514, .119) .232
**006_8**	-.078 (-.390, .236) .642	-.096 (-.399, .240) .566	-.043 (-.402, .311) .798	-.102 (-.508, .271) .542	.213 (-.100, .479) .200	-.932 (-.970, -.879) < .001***
006_9	-.052 (-.354, .264) .758	-.080 (-.399, .292) .635	-.047 (-.338, .278) .782	-.211 (-.489, .129) .204	.036 (-.243, .343) .829	-.306 (-.571, -.040) .061
006_10	.037 (-.248, .331) .825	-.065 (-.415, .258) .697	-.095 (-.392, .254) .570	-.201 (-.521, .162) .226	.127 (-.147, .405) .449	-.178 (-.471, .115) .285
006_12	-.046 (-.338, .311) .782	-.093 (-.410, .267) .579	-.046 (-.344, .300) .782	-.199 (-.467, .181) .231	.058 (-.263, .356) .731	-.295 (-.557, -.041) .072
006_13	-.077 (-.383, .255) .645	-.027 (-.354, .309) .874	.007 (-.315, .371) .968	-.112 (-.470, .267) .504	.072 (-.164, .339) .669	-.313 (-.590, -.021) .056
**006_14.15**	-.054 (-.359, .239) .746	-.109 (-.455, 236) .515	-.059 (-.381, .319) .723	-.188 (-.481, .175) .259	.120 (-.162, .407) .474	-.329 (-.579, -.063) .044*
**006_16**	-.050 (-.362, .278) .766	-.128 (-.450, .221) .445	-.019 (-.338, .308) .911	-.207 (-.494, .182) .212	.086 (-.222, .396) .609	-.337 (-.600, -.062) .039*
**006_avg**	-.048 (-.350, .270) .776	-.065 (-.403, .266) .697	-.040 (-.348, .276) .813	-.212 (-.531, .161) .201	.120 (-.175, .395) .474	-.411 (-.638, -.163) .010*
**008_3**	-.109 (-.370, .195) .528	-.253 (-.557, .125) .136	-.033 (-.269, .221) .850	-.160 (-.482, .206) .351	.168 (-.194, .469) .326	.557 (.278, .738) < .001***
008_4	.199 (-.053, .411) .238	.056 (-.368, .275) .742	-.086 (-.350, .401) .613	-.203 (-.412, .097) .229	-.027 (-.247, .198) .875	.103 (-.484, .381) .543
008_6	.144 (-.175, .413) .394	.110 (-.240, .443) .516	.082 (-.210, .429) .631	-.189 (-.471, .151) .262	.007 (-.275, .322) .968	-.009 (-.340, .297) .956
008_10	.110 (-.169, .397) .516	.088 (-.256, .389) .606	.054 (-.300, .343) .751	-.205 (-.566, .186) .223	-.008 (-.347, .314) .965	-.196 (-.519, .101) .245
**008_avg**	.078 (-.219, .352) .647	-.061 (-.375, .260) .718	-.011 (-.253, .293) .946	-.257 (-.578, .146) .125	.081 (-.197, .358) .632	.330 (-.035, .562) .046*
010_2	-.050 (-.390, .293) .765	.017 (-.351, .325) .919	-.170 (-.475, .154) .308	-.282 (-.595, .145) .086	.106 (-.228, .410) .527	-.228 (-.521, .043) .169
**010_3.4**	-.140 (-.41, .193) .401	-.021 (-.331, .301) .901	-.085 (-.411, .336) .612	-.202 (-.492, .196) .224	.094 (-.244, .385) .575	-.362 (-.605, -.073) .026*
010_6	-.154 (-.438, .207) .357	-.010 (-.352, .324) .954	-.146 (-.469, .173) .383	-.188 (-.506, .221) .259	.112 (-.223, .385) .502	-.317 (-.591, -.004) .053
**010_8**	-.159 (-.434, .166) .339	-.048 (-.381, .271) .775	-.194 (-.502, .171) .243	-.174 (-.494, .219) .295	.117 (-.229, .413) .482	-.336 (-.599, -.052) .039*
010_14^d^	-.145 (-.414, .177) .386	.004 (-.373, .325) .980	-.168 (-.483, .180) .313	-.218 (-.501, .156) .189	.040 (-.291, .414) .814	.241 (-.142, .546) .145
010_ avg	-.128 (-.432, .230) .444	-.015 (-.342, .296) .928	-.154 (-.495, .192) .356	-.220 (-.555, .182) .185	.110 (-.238, .395) .509	-.318 (-.587, -.031) .052

Pearson partial correlation coefficients were calculated using a bootstrapping procedure based on percent methylation values for COMT CpG units within the top 40^th^ percentile for differential methylation within the healthy human sample. Correlations in each column controlled for all other column variables. Results are based on *n* = 48 samples. AUDIT, Alcohol Use Disorders Identification Test; CI, confidence interval; SES, socioeconomic status. ^a^male = 0, female = 1; ^b^white = 1, non-white = 0 ^c^Not within 40^th^ percentile for differential methylation, but selected *a priori* based on [56]. ^d^Not within 40^th^ percentile for differential methylation, but selected *a priori* because CpG unit is located within the rs4680 SNP and present only in COMT^Val^ allele [33]. *p < .05, **p < .01, ***p < .001, **uncorrected for FWE**, ***FDR corrected (p < .05)***.

When considering Val allele carriers and Met allele carriers separately, we found significantly greater methylation in whites relative to non-whites at CpG 003_1 in both groups (both *p* = .009). In contrast, we observed significantly less methylation among whites at CpG 006_3 among Met carriers (*r* = -475, *p* = .026), but not among Val carriers (-.302, *p* = .105). In addition, significant hypomethylation of CpGs in amplicons 005, 006, 008, and 010 were observed among white relative to non-white Met carriers, but not Val carriers. In fact, we saw significant hypomethylation of each DM CpG in amplicon 010 among white relative to non-white Met carriers (010_avg: *r* = -.551, 95% CI: -.832, -.023, *p* = .008), which was not evident among Val carriers (010_avg: *r* = -.147, 95% CI: -497, .261, *p* = .439). The DM at CpG 001_34 associated with SES was also COMT allele-specific: we observed a significant association between SES and DM among Met carriers (*r* = -.447, 95% CI: -.721, -.025, *p* = .042), but not Val carriers (*r* = -.258, 95% CI: -.643, .261, *p* = .185). After accounting for the effects of other factors, we did not observe any significant correlations between methylation and age or sex specific to Val or Met carriers.

In addition, numerous studies have reported interactions between *Val*^*158*^*Met* genotype and environmental stressors, on risk of alcoholism [35]. Although data from human postmortem brain samples has shown less global DNA methylation in the brains of alcoholics relative to controls [67], we found no correlation between methylation and alcohol use in the sample as a whole when controlling for effects of the other factors. Among Met carriers, however, we did find significant hypomethylation of amplicon 001 with increasingly hazardous alcohol use (001_avg: *r* = -.435, 95% CI: -.728, -.114, *p* = .049); no such relationship was observed among Val carriers (001_avg: *r* = -.038, 95% CI: -.473, .381, *p* = .847). Qualitatively similar results were obtained when participants were dichotomized according to whether they were “possible hazardous drinkers” or not, based on AUDIT scores [60] (data not shown).

### Relationship between methylation of COMT and COMT gene expression in breast cancer cell lines

To evaluate the functional consequences of methylation at particular CpGs within *COMT*, we evaluated the effects of methylation on *COMT* expression in a sample containing both estrogen receptor positive and negative breast cancer cell lines by quantitative real-time reverse transcription-PCR (qRT-PCR). As expected, *COMT* DNA methylation in human cell lines was inversely correlated with *COMT* mRNA expression at several loci. Specifically, *COMT* gene expression was negatively correlated with percent methylation in all of the DM CpGs within amplicon 006 (see Table 4), including CpG 006_3 (*r* = -.683, 95% CI: -.820, .525, *p* = .029), a CpG within the exon containing both ATG translational start sites for both S- and MB-COMT, respectively (Figure 1) that was previously reported to be DM across individuals [56], and which was also DM within our saliva samples from healthy controls.

**Table 4:** Gene expression versus COMT methylation in human cell lines

**CpG units**	**Spearman’s *rho***	**95% C.I.**	***p value***
001_1	-.252	(-.923, .493)	.483
001_5	-.220	(-.886, .415)	.541
001_30	-.171	(-.889, .712)	.636
001_32	.020	(-.837, .808)	.956
001_33	-.049	(-.879, .698)	.894
001_avg	-.479	(-.950, .324)	.162
005_4	-.377	(-.808, .401)	.283
005_7	-.455	(-.920, .358)	.187
005_8	-.198	(-.863, .519)	.584
005_9	-.127	(-.801, .680)	.726
005_10	-.206	(-.808, .615)	.567
005_11	-.105	(-.801, .699)	.773
005_12	-.334	(-.849, .472)	.345
005_17	-.079	(-.892, .690)	.828
005_18	-.421	(-.919, .377)	.226
005_avg	-.333	(-.796, .405)	.347
**006_3**	**-.683**	**(-.820, -.525)**	.029*
**006_5**	**-.806**	**(-.968, -.245)**	.005**
**006_9**	**-.782**	**(-.988, -.305)**	.008**
**006_12**	**-.733**	**(-.987, -.119)**	.016*
**006_13**	***-.860***	***(-.990, -.472)***	.001**
**006_avg**	**-.766**	**(-.963, -.082)**	.010*

Spearman correlation coefficients were calculated using a bootstrapping procedure based on log relative gene expression between ABI TaqMan probes for COMT and an 18S rRNA endogenous control with percent methylation values for CpG units in the top 40^th^ percentile for differential methylation within the cell line samples. Methylation of COMT within amplicon 006 was significantly inversely correlated with COMT expression, with coefficients ranging from -0.683 to -0.860 (*p* = 0.029–0.001). Results are based on *n* = 10 tumors for which there were both complete methylation and gene expression data. * ***p*** **< .05**, ** ***p*** **< .01**, *** ***p*** **< .001**, **uncorrected for FWE**, ***FDR corrected (p < .05)***.

## Discussion

We studied DNA methylation throughout the large *COMT* gene, interrogating over 200 CpG dinucleotides spanning more than 27,000 base pairs. To our knowledge, this is the first comprehensive assessment of *COMT* DNA methylation, and also the most comprehensive test of associations between such methylation and non-genetic factors, the *Val*^*158*^*Met* genotype, and their interactions. With the exception of the hypomethylated amplicon 001 covering part of the 5’UTR and exon 1 of *COMT* (Figure 1), the remaining 12 amplicons were relatively hypermethylated, with an observed dynamic range of methylation typically between 50–90 percent.

By employing the use of the EpiTYPER^®^ MassARRAY platform to precisely quantify methylation for each CpG unit, we were able to pinpoint specific, differentially methylated CpG sequences that were highly informative between sample types and by subject features. Indeed, Figures 3B-C underscore the importance of consecutive interrogation of CGs throughout CpG islands in that we were able to identify transitional CpGs in amplicons 005 and 006 where the balance of methylation shifted from a methylated to a relatively unmethylated state. Notably, we identified significant associations with COMT Met alleles in these transitional regions of both amplicons 005 and 006. Additionally, we identified what, to our knowledge, is a newly described ~40 bp repeat sequence motif that begins in exon 1-v2. This repeat motif spans ~1,500 bp, and given its exonic location, is of potential significance in that it may have an effect on MB-COMT expression. What is certain, however, is that this repeat motif made PCR amplification of the COMT_002 amplicon challenging, and rendered amplification of COMT_004 nearly impossible due to stuttering of Taq polymerase through multiple repeats over large bp distances. Thus the DM within some regions of *COMT* remains unknown, although future studies may be able to overcome this technical challenge.

Although the size of our sample of salivary DNA from a community sample of self-identified social drinking adults, albeit representing a continuum from light to heavy drinkers, was not large (*n* = 48), we note that these data replicate the previously reported finding of significant DM in the putative *COMT* promoter region (designated amplicon COMT_006 here, particularly 006_3) [33,56]. Like Ursini and colleagues, we found an effect of *Val*^*158*^*Met* genotype on methylation in this promoter region, however, that study found increasing methylation with Met allele number, whereas we observed reduced methylation in white samples that was present only in Met carriers, but no independent effect of Met alleles. Discrepancies may reflect sample differences: Ursini et al.’s sample was all white and predominantly female, whereas ours was ethnically mixed and half female. It could also reflect analytical strategy differences, as we assayed at the level of individual CpGs, and evaluated partial correlations, which controlled for effects of other factors, while Ursini et al. did not. Our 006_avg results may be the closest measure to theirs, and while we did find a significant effect of *Val*^*158*^*Met* on methylation of 006_avg, our observed relationship was in the opposite direction (less methylation with more Met alleles). Future work with larger samples allowing for well-powered stratification for sex and ethnicity may help resolve this issue. The robustness of the finding of DM in the COMT_006 region, including here in two small independent samples, coupled with our novel finding of significant associations between COMT_006 methylation with *COMT* expression highlights the importance of further work in this area. We also report novel associations between methylation in COMT_001 with SES, and COMT_003, 005, and 006 with ethnicity (Table 3), with many of these effects interacting with *Val*^*158*^*Met* genotype. These latter findings indicate the importance of controlling for these factors in future studies. Viewed in light of reported associations with stress and both race/ethnicity and SES (e.g. [68]), and the numerous recent reports of interacting effects of stress and *Val*^*158*^*Met* genotype on a large variety of conditions [33-38], differential *COMT* methylation state as a function of stress in Met carriers is a plausible underlying molecular mechanism for these interacting associations and warrants further study.

The relationship between DNA methylation and alcohol use in human samples is a topic of growing interest, although to date, our understanding is far from complete [39]. For example, less global DNA methylation is observed in postmortem brain samples from alcoholics relative to controls [67]. In contrast, samples from living human subjects have found reduced global methylation among ever-drinkers relative to non-drinkers [69], elevated global methylation among alcoholics compared with controls [70], or no differences in DNA methylation based on alcohol use [71,72]. Studies investigating sites within candidate genes have shown mixed results, although together, findings suggest associations between DNA methylation and alcohol use or alcohol use disorders in human samples, although the relationships appear complex [39]. To our knowledge, only two such studies have interrogated CpGs within the COMT gene [73,74], which both interrogated 3 CpGs in the promoter region (along with 381 other CpGs in other candidate genes), finding no association between these three sites and alcohol use. However, as we found here, such associations may interact with COMT genotype, which was not accounted for in those studies. Specifically, we found significant hypomethylation of amplicon 001 with increasingly hazardous alcohol use only among Met carriers. Notably, we also observed significant hypomethylation at a locus within amplicon 001 with lower SES specific to Met carriers. Given the finding that Met carriers show increasing risk of alcoholism with greater childhood adversity (which negatively correlates with SES) [35], it is tempting to speculate that this gene × environment interaction could derive in part from additive hypomethylation in the promoter regions of the COMT gene in Met carriers as a consequence of both childhood adversity and high alcohol intake. Further work investigating the functional consequences of such methylation is needed, although our gene expression data showed a trend toward decreased *COMT* expression with increasing methylation. Thus, hypomethylation would be predicted to result in increased COMT expression, reducing tonic levels of frontal dopamine in Met carriers.

### Study limitations

As noted above, amplicons COMT_005 and COMT_006 overlap or flank either a predicted or known promoter of S-COMT. While COMT_006 methylation was strongly inversely correlated with *COMT* expression, one limitation of our study is that we did not have corresponding RNA from our healthy adult saliva-derived DNA samples with which to perform RT-PCR, nor did the commercially available COMT TaqMan probe we used distinguish between S- and MB-COMT transcripts in our breast cancer cell lines. Therefore, our correlations with COMT expression and methylation at specific COMT CpG loci in breast cell lines may not generalize to any other tissue or sample type. Future studies employing the 5’-rapid amplification of cDNA ends (RACE) method to distinguish between methylation of distal and proximal promoters and expression of both S- and MB-COMT transcripts in multiple human tissues in both affected and healthy individuals will help resolve the relationship between *COMT* methylation at particular loci and expression of distinct transcript variants [40]. Some previous work has also reported DM of *COMT* in the region upstream from MB-COMT Exon 1, with variation associated with *Val*^*158*^*Met* genotype as well as disease states [57,58]. We did not fully interrogate this region, but future work using the EpiTYPER^®^ MassARRAY platform to precisely quantify methylation for each CpG unit in that region may prove fruitful.

With the exception of the proximal promoter and Exon 1, COMT is a highly methylated gene with a dynamic range of methylation between 50-100%. Overall, our findings suggest that regulation of *COMT* expression is highly complex, and likely hinges on multiple factors and interactions including the *Val*^*158*^*Met* genotype, methylation of several loci, and a host of other biological and environmental factors. Future work will employ more specific gene expression probes for the S- and MB-COMT transcripts, together with mechanistic association studies of locus specific DNA methylation with transcription factor binding and higher chromatin configurations with COMT.

## Conclusions

In summary, we report the first comprehensive analysis of DM within the *COMT* gene using precisely quantified methylation for each CpG unit. In doing so, we have pinpointed specific, differentially methylated CpG sequences that were highly informative between sample types and by individual differences between samples. Some of these findings solidify existing literature, while other data identifies novel sites at which regulation of *COMT* expression may be achieved by specific biological or environmental factors. Given the broad function of *COMT* and its implication in a wide spectrum of clinical conditions, a thorough understanding of how expression of this gene is regulated may be therapeutically transformative in diseases as diverse as alcoholism and breast cancer. The present findings make a substantial contribution in that direction.

## Competing interests

The authors declare that they have no competing interests.

## Authors’ contributions

TS-S participated in the design of the study, coordinated and carried out the molecular studies, conducted the methylation and cluster analyses, and drafted the manuscript. CTS extracted DNA from saliva samples and compiled participant data. SAB helped with the gene expression assays, and participated in gene annotation and sequence alignment. CAB conceived of the study, participated in the design of the study, performed the other statistical analyses and drafted the manuscript. All authors read and approved the final manuscript.

## Pre-publication history

The pre-publication history for this paper can be accessed here:

http://www.biomedcentral.com/1755-8794/7/5/prepub
